# Value of Medical Practice in London

**Published:** 1847-08

**Authors:** 


*Value of Medical Practice in London*—Thirty Surgeons of London have sworn that their annual income amounted to $50,000, each, and three to upwards of $100,000. Sir Astley Cooper's business in his best days about $115,000 per annum.

